# Venetoclax in combination with carfilzomib and dexamethasone in relapsed/refractory multiple myeloma harboring t(11,14)(q13;q32): two case reports and a review of the literature

**DOI:** 10.1186/s13256-020-02376-y

**Published:** 2020-04-23

**Authors:** Khadega A. Abuelgasim, Noha Alherz, Ayman Alhejazi, Moussab Damlaj

**Affiliations:** 1 King Abdullah International Medical Research Center, Riyadh, 11481 Saudi Arabia; 2 Oncology Department, King Abdulaziz Medical City, Ministry of National Guard Health Affairs, Riyadh, 11426 Saudi Arabia; 3 King Saud bin Abdulaziz University for Health Sciences, Riyadh, 14611 Saudi Arabia

**Keywords:** Multiple myeloma, Relapsed/refractory, t(11:14)(q13;q32), Venetoclax

## Abstract

**Background:**

Multiple myeloma has witnessed significant advances due to the approval of many novel agents. However, in spite of all these new developments, multiple myeloma remains an incurable disease with inevitable relapse in the majority of patients. Venetoclax is a selective antiapoptotic protein B-cell lymphoma 2 inhibitor that induces cell death in multiple myeloma cells, particularly in those harboring t(11,14)(q13;q32). We report two cases of patients with multiple myeloma with t(11,14)(q13;q32) who were treated with venetoclax/carfilzomib/dexamethasone with rapid initial response; however, the response was short-lived.

**Cases presentation:**

Patient 1 was a 50-year-old Saudi man with International Staging System stage III kappa light chain multiple myeloma with normal karyotype diagnosed in May 2013. He received bortezomib/thalidomide/dexamethasone treatment and underwent autologous hematopoietic stem cell transplant. Three years later, he presented with disease progression and received multiple lines of chemotherapy, including carfilzomib/lenalidomide/dexamethasone. Venetoclax/carfilzomib/dexamethasone was started after acquiring t(11,14)(q13;q32) 5 years into his disease course. He achieved complete remission, with disease progression after cycle 6. Patient 2 was a 48-year-old Saudi man with International Staging System stage III immunoglobulin G kappa multiple myeloma with t(11,14)(q13;q32) diagnosed in May 2017. He received bortezomib/thalidomide/dexamethasone treatment and underwent autologous hematopoietic stem cell transplant. Eighteen months later, he had disease progression, and he received multiple lines of chemotherapy, including carfilzomib/dexamethasone. He was shifted to venetoclax/carfilzomib/dexamethasone in April 2019 and had an initial clinical response; two months later, he progressed to plasma cell leukemia with rapid deterioration to multiorgan failure.

**Conclusions:**

Acquired t(11;14)(q13;q32) is unreported in the multiple myeloma literature. In the era of targeted therapy, it is essential to repeat the cytogenetic and multiple myeloma fluorescence *in situ* hybridization panel with each disease progression. Multiple myeloma remains a challenging hematological malignancy despite advances in personalized/precision medicine.

## Background

Multiple myeloma (MM) has witnessed significant advances over the last two decades with regard to diagnostic methodologies and approval of many novel agents that have significantly prolonged the survival of patients. However, in spite of all these new developments, MM remains an incurable disease with inevitable relapse in the majority of patients [1]. Cytogenetic aberrations are quite common in MM, and they are used as prognostic factors to estimate the outcome of patients. t(11,14)(q13;q32) is a commonly detected aberration; it is found in up to 20% of newly diagnosed patients with MM. Although the presence of t(11,14)(q13;q32) stratifies patients as having standard risk disease, the response rates and overall outcomes in these patients appear to be inferior to those of their other standard risk counterparts [2–5]. Typically, t(11,14)(q13;q32) has been associated with lymphoplasmacytic morphology and increased number of circulating plasma cells (PCs) [6]. Furthermore, it results in the upregulation of cyclin D1 and antiapoptotic protein B-cell lymphoma 2 (Bcl-2), thus raising the hypothesis of whether Bcl-2 inhibition could be a potential target in the treatment of t(11,14)(q13;q32) MM [7].

Venetoclax is the first-in-class selective Bcl-2 inhibitor (Bcl-2-specific BH3 mimetic) that induces cell death in MM cells, particularly in those patients harboring t(11,14)(q13;q32). Venetoclax monotherapy has demonstrated efficacy in the treatment of patients with relapsed/refractory multiple myeloma (RRMM) [8]. Venetoclax-containing combinations with dexamethasone with or without bortezomib were found to be of even higher efficacy [9].

BELLINI (A Study Evaluating Venetoclax [ABT-199] in Multiple Myeloma Subjects Who Are Receiving Bortezomib and Dexamethasone as Standard Therapy) is a phase III, double-blind, multicenter trial investigating bortezomib/dexamethasone (Bd) plus venetoclax (VenBd) vs. Bd placebo in patients with RRMM. Eleven percent of the patients enrolled in the venetoclax arm had t(11,14)(q13;q32), as compared with 16% in the placebo arm. Following a median follow-up of 18.7 months, the overall response rate (ORR) was 82% in the venetoclax arm compared with 68% in the placebo arm. However, the rate of death was 21.1% (41 of 194 patients) in the venetoclax arm compared with 11.3% (11 of 97 patients) in the placebo arm. Eight of the 13 treatment-related deaths in the venetoclax arm were attributed to infection. The median overall survival was not reached (hazard ratio, 2.027; 95% confidence interval, 1.042–3.945). Subgroup analysis of patients with t(11,14)(q13;q32) benefited the most from VenBd without treatment-related toxicity [10]. In March 2018, the U.S. Food and Drug Administration issued a partial clinical hold of enrollment of new patients with MM in venetoclax trials, pending further review of these results.

In a phase II dose escalation study, the combination of venetoclax, carfilzomib, and dexamethasone (VenKd) was tested in 42 patients with RRMM; 19% of them had t(11,14)(q13;q32). The ORR was 78% with very good partial remission (VGPR) or better seen in 56% of the whole cohort. Interestingly, the ORR was 100% with VGPR or better of 88% in the among patients with t(11;14)(q13;q32) with no new safety signals [11]. In this report, we describe two cases of patients with RRMM harboring t(11,14)(q13;q32) who received 7 and 10 total lines of therapy, respectively, with short-lasting response to VenKd.

## Case presentations

### Patient 1

A 50-year-old Saudi man who was diagnosed with International Staging System (ISS) stage III kappa light chain MM presented with acute kidney injury (serum creatinine 309 μmol/L), hypercalcemia (corrected serum calcium 3.15 mmol/L), and anemia (hemoglobin 119 g/L) in July 2013. The results of serum and urine protein electrophoresis were both negative for M protein spike. Serum free light chain showed kappa of 1830 mg/L and lambda of 10.1 mg/L with a kappa/lambda (K/L) ratio of 180.7. Bone marrow aspiration and biopsy (BMBx) showed diffuse infiltration by kappa restricted PCs; bone marrow cytogenetics were consistent with normal male karyotype. The result of fluorescence *in situ* hybridization (FISH) for MM recurrent alterations, including t(11,14)(q13;q32), was negative.

The patient was initially treated with bortezomib/thalidomide/ dexamethasone (VTd) for a total of five cycles, and he achieved a complete response (CR). He underwent autologous hematopoietic stem cell transplant (auto-HSCT) with 140 mg/m^2^ melphalan conditioning due to his dialysis-dependent end-stage renal disease. Three months following auto-HSCT, two cycles of consolidative VTd were given, followed by maintenance lenalidomide 5 mg orally every other day. He came off of dialysis 1 year after undergoing auto-HSCT.

Two years following auto-HSCT, he presented with bony pain. His serum kappa was 1000 mg/L and lambda was 19.9 mg/L, with K/L ratio of > 19.9. Positron emission tomography with computed tomography (PET/CT) revealed multiple new bony lesions. The result of BMBx was consistent with 32% kappa restricted PCs, all collectively consistent with progressive disease (PD). He was salvaged with three cycles of carfilzomib/lenalidomide/dexamethasone (KRd). He was refractory to KRd because he presented with bony pain and rising kappa 31,840.00 mg/L and lambda 5.00 mg/L, with K/L ratio of 6368 and evidence of new lesions seen by PET/CT. Due to multiple vertebral compression fractures and intractable pain, he underwent vertebroplasty in October 2016, and his therapy was switched to bortezomib/cyclophosphamide/dexamethasone, and he received a total of three cycles, achieving very good partial response (VGPR) with kappa 1250.00 mg/L and lambda 156.41 mg/L with a K/L ratio of 157.23.

To deepen his response in preparation for a second auto-HSCT, he was started on 25% dose-reduced bortezomib/dexamethasone/cisplatin/doxorubicin/cyclophosphamide, achieving VGPR after two cycles with less than 5% PCs in BMBx; negative PET/CT results; and serum kappa 302.00 mg/L, lambda 2.16 mg/L, with K/L ratio 139.81.

He subsequently underwent a second auto-HSCT 1 year following his first relapse. Assessment on day + 90 after auto-HSCT showed kappa 63.70 mg/L, lambda 13.70 mg/L, with K/L ratio of 4.6. The result of PET/CT was negative for active disease. He was started on bortezomib maintenance therapy every other week.

In March 2018, he presented with biochemical relapse while receiving bortezomib maintenance therapy with kappa 17,400.00 mg/L, lambda 20.40 mg/L, with K/L ratio of 85.29, but no evidence of active disease on a PET/CT scan. His estimated creatinine clearance was < 30 mmol/L, so he was considered ineligible for allogeneic stem cell transplant and therefore was started on elotuzumab/lenalidomide/dexamethasone, of which he received a total of five cycles until July 2018. On August 18, 2018, he presented with bony pain and was found to have multiple new lytic lesions and multiple pathological fractures with kappa 9650.00 mg/L, lambda 9.78 mg/L, with K/L ratio > 986.7 consistent with PD; therefore, he was started on daratumumab/vincristine/dexamethasone for a total of six cycles. Evaluation after the sixth cycle showed kappa 565.00 mg/L, lambda 5.92 mg/L, with K/L ratio of 954.39. BMBx showed cellularity 20% with only 5% PCs, but with newly acquired t(11,14)(q13;q32).

On March 16, 2019, he was started on venetoclax/carfilzomib/dexamethasone (VenKd). The dose of carfilzomib was 56 mg/m^2^ on days 1, 8, and 22; dexamethasone 40 mg weekly; and venetoclax was started at 50 mg per day, escalated to 200 mg per day over a period of 2 months. However he required multiple dose adjustments due to myelosuppression. Assessment after three cycles of VenKd included BMBx, which showed 40–50% cellularity with 1% PCs and serum kappa 492.50 mg/L, lambda 7.21 mg/L, with K/L ratio of 68.1. PET/CT showed no evidence of PET/CT-avid lesions consistent with VGPR. A repeat PET/CT scan after six cycles of VenKD was consistent with PD because it showed multiple new avid lesions and serum kappa 580.00 mg/L, lambda 7.38 mg/L, with K/L ratio of 78.59. The patient was started on cyclophosphamide/carfilzomib/dexamethasone and has received three cycles thus far. The evolution of therapy in patient 1 is shown in Table 1.

**Table 1:** Evolution of therapy in patient 1

Regimen	Duration of therapy (mo)	Best response	Reason for stopping
Bortezomib/thalidomide/dexamethasone (VTd)	5 (Aug–Dec 2013)	CR	Auto-HSCT
VTd consolidation	2 (Jun–Jul 2014)	CR	Started maintenance
Lenalidomide maintenance	18 (Aug 2014–Jan 2016)	CR	PD
Carfilzomib/lenalidomide/dexamethasone (KRd)	3 (March–May 2016)	PD	PD
Bortezomib/cyclophosphamide/dexamethasone (VCd)	3 (Aug–Oct 2016)	PR	PD
Bortezomib/dexamethasone/cisplatin/doxorubicin/cyclophosphamide (DV-PACE)	2 (Dec 2016–Jan 2017)	VGPR	Auto-HSCT
Bortezomib maintenance	11 (Apr 2017–Feb 2018)	VGPR	PD
Elotuzumab/lenalidomide/dexamethasone (ERd)	5 (Mar–Aug 2017)	PD	PD
Daratumumab/bortezomib/dexamethasone (DVd)	6 (Sep 2017–Feb 2018)	VGPR	t(11;14) acquired
Venetoclax/carfilzomib/dexamethasone (VenKd)	6 (Mar–Sep 2019)	CR	PD
Cyclophosphamide/carfilzomib/dexamethasone (CKd)	2 (Oct 2019–present)	NA	NA

*Abbreviations: Auto-HSCT* autologous hematopoietic stem cell transplant, *CR* complete remission, *NA* not applicable, *PD* progressive disease, *PR* partial remission, *VGPR* very good partial remission

### Patient 2

A 48-year-old Saudi man was diagnosed with ISS stage III immunoglobulin G kappa MM in May 2017 when he presented with bony pain and was found to have multiple lytic lesions by PET/CT. His BMBx revealed 80% PCs, and FISH analysis showed isolated t(11,14)(q13;q32). His serum kappa was 584.00 mg/L, lambda was 2.54 mg/L, and K/L ratio was 229.92.

He was started on bortezomib/lenalidomide/dexamethasone (VRd). Lenalidomide was replaced by thalidomide (VTd) due to his severe allergic reaction in the first cycle. Assessment after four cycles of VTd showed serum kappa 9.58 mg/L, lambda 11.00 mg/L, and K/L ratio of 0.87, and BMBx showed PCs of < 1%, consistent with stringent complete remission. On October 11, 2017, he underwent auto-HSCT. Assessment on day + 90 after auto-HSCT showed morphological remission in bone marrow and serum kappa 13.00 mg/L, lambda 8.32 mg/L, and K/L ratio of 1.56, consistent with maintained response. Three months after auto-HSCT, he was started on thalidomide maintenance due to lenalidomide allergy. He developed grade 3 neurotoxicity despite dose adjustments; therefore, thalidomide was discontinued after 8 months.

On November 25, 2018, he presented with clinical PD in the form of extramedullary plasmacytomas in the left thumb and middle finger and serum kappa of 285.00 mg/L, lambda of 8.63 mg/L, and K/L ratio of 32.80. His PET/CT scan showed new active bony lesions consistent with PD. He was started on Kd, which was complicated by intensive care unit admission due to respiratory failure and acute kidney injury requiring hemodialysis.

On February 11, 2019, he was started on daratumumab/pomalidomide/dexamethasone; he received a total of two cycles with evidence of PD with worsening of plasmacytomas in his fingers as well as a new nasal mass associated with pain and epistaxis. Because the patient had t(11,14)(q13;q32), he was shifted to VenKD on April 8, 2019. Venetoclax was dosed at 400 mg per day, then escalated to 600 mg per day over a period of 1 month; carfilzomib was dosed at 56 mg/m^2^ on days 1, 8, and 22 and dexamethasone at 40 mg weekly. Marked initial clinical response was observed. However, on June 8, 2019, after the third VenKd cycle, he presented with generalized body weakness, and his creatinine increased to 449 μmol/L from baseline of 69 μmol/L; his white blood cell count increased to 34,000 × 10^9^/L from a baseline of 7.41 × 10^9^/L; his blood smear showed 30% PCs; and BMBx showed hypercellularity of 90% with diffuse infiltration of PCs consistent with PC leukemia. His PET/CT scan showed multiple new active bony lesions consistent with PD. His general condition rapidly deteriorated, and ultimately he died of multiorgan failure on June 27, 2019. The evolution of therapy in patient 2 is shown in Table 2.

**Table 2:** Evolution of therapy in patient 2

Regimen	Duration of therapy (mo)	Best response	Reason of stopping
Bortezomib/lenalidomide/dexamethasone (VRd)	1 (May 2017)	NA	Allergic reaction
Bortezomib/thalidomide/dexamethasone (VTd)	4 (Jun–Aug 2017)	CR	Auto-HSCT
Thalidomide maintenance	8 (Feb–Sep 2018)	VGPR	PD
Carfilzomib/dexamethasone (Kd)	2 (Dec 2018–Jan 2019)	NA	Respiratory failure
Daratumumab/pomalidomide/dexamethasone (DPd)	2 (Feb–Mar 2019)	PD	PD
Venetoclax/carfilzomib/dexamethasone (VenKd)	3 (Apr–Jun 2019)	Improvement of plasmacytoma	Plasma cell leukemia

*Abbreviations: Auto-HSCT* autologous hematopoietic stem cell transplant, *CR* complete remission, *NA* not applicable, *PD* progressive disease, *PR* partial remission, *VGPR* very good partial remission

## Discussion and conclusions

Different subpopulations of patients with MM have different outcomes driven by well-characterized genetic abnormalities that occur at various time points of the disease course [12, 13]. Specific cytogenetic abnormalities in MM affect clinical presentation, prognosis, and more recently management strategies. The t(11;14) is a common primary cytogenetic abnormality in MM. Patients with MM who have t(11;14) tend to present with more bone disease and are considered to be at standard risk with a median overall survival of 7–10 years [14, 15]. Both of our patients presented with bony lesions; although one is still alive more than 6 years after diagnosis, the other died within 2 years of diagnosis.

Among the known secondary cytogenetic abnormalities that are acquired during the course of disease, usually with disease progression, are t(4;14), t(14;20), t(14;16), Myc translocation, 1q amplification, 17p deletion, and 1p deletion [16]. The result of patient 1’s diagnostic FISH was negative for the known myeloma-related cytogenetic abnormalities; he subsequently acquired t(11;14)(q13;q32) 5 years into his disease course, which had been unreported in the MM literature to date. In the era of targeted therapy, it is essential to repeat the MM FISH panel with each disease progression to allow precision therapy.

Patients with MM who have t(11;14)(q13;q32) are known to have an excellent response to lenalidomide-based therapy [17]. Our patient 1 received multiple lenalidomide-based regimens and is still alive more than 6 years after diagnosis, whereas our patient 2 could not receive lenalidomide due to extreme allergy, which might have contributed to his worse outcome and shorter survival.

Venetoclax combinations, including VenKd, showed promising preliminary results in patients with RRMM in phase Ib and phase II studies; however, all of these studies had short follow-up [9–11]. Venetoclax in patients with MM harboring t(11,14)(q13;q32) is considered the first example of personalized/precision therapy in the field of MM; however, more clinical studies with longer follow-up are needed before its adoption in clinical practice. Both of our patients had disease that failed to respond to multiple regimens, including those containing carfilzomib and dexamethasone. In both patients, VenKD resulted in a rapid initial response; however, one patient’s disease progressed after 6 months, whereas the other’s progressed after only 2 months.

MM remains a challenging hematological malignancy that we fail to cure despite advances in personalized/precision medicine. In the era of targeted therapy, it is essential to repeat the MM FISH panel with each disease progression to allow for precision therapy. The use of novel MM therapeutic combinations is better done within a clinical trial.

## Data Availability

The data used in this report are available from the corresponding author on reasonable request.

## Abbreviations

Auto-HSCT: Autologous hematopoietic stem cell transplant
Bcl-2: Antiapoptotic protein B-cell lymphoma 2
Bd: Bortezomib/dexamethasone
BMBx: Bone marrow aspiration and biopsy
CR: Complete remission
FISH: Fluorescence *in situ* hybridization
K/L: Kappa/lambda
ISS: International Staging System
Kd: Carfilzomib/dexamethasone
KRd: Carfilzomib/lenalidomide/dexamethasone
MM: Multiple myeloma
ORR: Overall response rate
PD: Progressive disease
PC: Plasma cell
PET/CT: Positron emission tomography with computed tomography
PR: Partial remission
RRMM: Relapsed/refractory multiple myeloma
VCd: Bortezomib/cyclophosphamide/dexamethasone
VenBd: Venetoclax/bortezomib/dexamethasone
VenKD: Venetoclax/carfilzomib/dexamethasone
VGPR: Very good partial remission
VTd: Bortezomib/thalidomide/dexamethasone
VRd: Bortezomib/lenalidomide/dexamethasone
